# Questionnaire discrimination: (re)-introducing coefficient *δ*

**DOI:** 10.1186/1471-2288-7-19

**Published:** 2007-05-18

**Authors:** Matthew Hankins

**Affiliations:** 1 King's College London, Department of Psychology (at Guy's), Institute of Psychiatry, London, UK; 2 Department of Primary Care & Public Health, Brighton & Sussex Medical School, Brighton, UK; 3 Brighton & Sussex University Hospitals NHS Trust, Royal Sussex County Hospital, Brighton, UK

## Abstract

**Background:**

Questionnaires are used routinely in clinical research to measure health status and quality of life. Questionnaire measurements are traditionally formally assessed by indices of reliability (the degree of measurement error) and validity (the extent to which the questionnaire measures what it is supposed to measure). Neither of these indices assesses the degree to which the questionnaire is able to discriminate between individuals, an important aspect of measurement. This paper introduces and extends an existing index of a questionnaire's ability to distinguish between individuals, that is, the questionnaire's discrimination.

**Methods:**

Ferguson (1949) [1] derived an index of test discrimination, coefficient *δ*, for psychometric tests with dichotomous (correct/incorrect) items. In this paper a general form of the formula, *δ*_*G*_, is derived for the more general class of questionnaires allowing for several response choices. The calculation and characteristics of *δ*_*G *_are then demonstrated using questionnaire data (GHQ-12) from 2003–2004 British Household Panel Survey (N = 14761). Coefficients for reliability (*α*) and discrimination (*δ*_*G*_) are computed for two commonly-used GHQ-12 coding methods: dichotomous coding and four-point Likert-type coding.

**Results:**

Both scoring methods were reliable (*α *> 0.88). However, *δ*_*G *_was substantially lower (0.73) for the dichotomous coding of the GHQ-12 than for the Likert-type method (*δ*_*G *_= 0.96), indicating that the dichotomous coding, although reliable, failed to discriminate between individuals.

**Conclusion:**

Coefficient *δ*_*G *_was shown to have decisive utility in distinguishing between the cross-sectional discrimination of two equally reliable scoring methods. Ferguson's *δ *has been neglected in discussions of questionnaire design and performance, perhaps because it has not been implemented in software and was restricted to questionnaires with dichotomous items, which are rare in health care research. It is suggested that the more general formula introduced here is reported as *δ*_*G*_, to avoid the implication that items are dichotomously coded.

## Background

Questionnaire measures are routinely used in clinical research as measures of health status and quality of life [2] as well as other outcomes such as mood, stress, satisfaction and so on. The theory underlying the use of questionnaires as instruments of measurement is predominantly psychometric [3], and in keeping with this tradition the measurement properties of such questionnaires are reported as indices of reliability and validity. The reliability coefficient (for example, Cronbach's *α*) estimates the degree of measurement error in the data, and hence the reproducibility of the measurements. Validity refers to the degree to which the questionnaire measures what is intended to be measured, and this is usually inferred from the degree to which the questionnaire agrees with other criteria.

Reliability and validity of measurement are of course paramount for good-quality data, but the degree to which a measurement instrument is capable of discerning differences between individuals is also a fundamental aspect of measurement theory [4]. For a questionnaire to be useful in assessing health status, it must be able to distinguish between individuals who differ in health status, and fail to distinguish between those who do not. A questionnaire that failed to distinguish real differences would be unlikely to be valid, and hence discrimination is a necessary but not sufficient condition of validity. The concept described here as 'discrimination' is also referred to as 'discriminatory power' [3] but should not be confused with discriminant validity, item discrimination or discriminant functions.

A little-reported statistic, Ferguson's [1]*δ*, quantifies the extent to which a measure can distinguish between cases. The statistic is conceptually simple. It is the ratio of observed differences to the theoretical maximum possible number of differences. When all possible scores occur with the same frequency, then the scale is maximally discriminating and the index is 1.0. Ferguson demonstrated that a normal distribution of test scores would yield a coefficient of around 0.9, and a rectangular distribution, 1.0. Skewed distributions result in fewer discriminations and hence lower values of *δ*, reaching a minimum of 0.0 when no discriminations at all are made and every respondent has the same score.

That this statistic has not been more widely used may be due to the limiting assumption that the measure comprises *dichotomous *items (e.g. incorrect/correct), with each response coded as 0 or 1. Most health status questionnaires use polytomous scales, typically five- or seven-point Likert-type scales (e.g. Strongly disagree, Disagree, Not sure, Agree, Strongly Agree). Researchers wishing to compute *δ *would therefore be forced to dichotomise item responses in order to compute the statistic.

As noted above, discrimination does not ensure validity: a high *δ *indicates that something is being discriminated, but not necessarily the thing intended. As Guilford [5] points out, any discussion of discrimination must take place within the more problematic context of validity. Interestingly, Guilford also suggested that the goals of maximising both discrimination and reliability may be incompatible. High reliability is sometimes claimed when the measure is constructed of highly-correlated items. As well as potentially limiting the validity of the resulting scale by excluding uncorrelated but valid items, this will tend to *decrease *discrimination. Depending on the circumstances it may be desirable to improve discrimination by increasing the heterogeneity of the questionnaire items at the cost of reliability (although reliability should not fall below an acceptable level). Hence discrimination should be a key consideration of questionnaires at the design stage.

The remainder of this paper develops the original formula for *δ *to allow for the computation of the statistic for questionnaire measures with polytomous items. The resulting general formula applies equally well to dichotomous and polytomous scales. The utility of the statistic will then be demonstrated using data from the 12-item General Health Questionnaire (GHQ-12) [6], which may be coded as the sum of 12 dichotomous items (known as 0011 coding) or of 12 items with four response categories (known as 0123 coding).

## Methods

Ferguson's formula for *δ *assumes that the test comprises one or more items, each with only two response categories: incorrect or correct. The items are therefore dichotomous and coded as 0 or 1, respectively. The definitional formula for *δ *is:

δ=n2−∑i=0kfi2n2−n2k+1
 MathType@MTEF@5@5@+=feaafiart1ev1aaatCvAUfKttLearuWrP9MDH5MBPbIqV92AaeXatLxBI9gBaebbnrfifHhDYfgasaacH8akY=wiFfYdH8Gipec8Eeeu0xXdbba9frFj0=OqFfea0dXdd9vqai=hGuQ8kuc9pgc9s8qqaq=dirpe0xb9q8qiLsFr0=vr0=vr0dc8meaabaqaciaacaGaaeqabaqabeGadaaakeaaiiGacqWF0oazcqGH9aqpdaWcaaqaaiabd6gaUnaaCaaaleqabaGaeGOmaidaaOGaeyOeI0YaaabCaeaacqWGMbGzdaqhaaWcbaGaemyAaKgabaGaeGOmaidaaaqaaiabdMgaPjabg2da9iabicdaWaqaaiabdUgaRbqdcqGHris5aaGcbaGaemOBa42aaWbaaSqabeaacqaIYaGmaaGccqGHsisldaWcaaqaaiabd6gaUnaaCaaaleqabaGaeGOmaidaaaGcbaGaem4AaSMaey4kaSIaeGymaedaaaaaaaa@46F1@

In which: *n *= sample size

*f *= frequency of score *i*

*k *= number of questionnaire items

This definitional formula has been further modified [5,7]. Guilford simplifies it to a computational formula as follows [5]:

δ=(k+1)(n2−∑i=0kfi2)kn2
 MathType@MTEF@5@5@+=feaafiart1ev1aaatCvAUfKttLearuWrP9MDH5MBPbIqV92AaeXatLxBI9gBaebbnrfifHhDYfgasaacH8akY=wiFfYdH8Gipec8Eeeu0xXdbba9frFj0=OqFfea0dXdd9vqai=hGuQ8kuc9pgc9s8qqaq=dirpe0xb9q8qiLsFr0=vr0=vr0dc8meaabaqaciaacaGaaeqabaqabeGadaaakeaaiiGacqWF0oazcqGH9aqpdaWcaaqaaiabcIcaOiabdUgaRjabgUcaRiabigdaXiabcMcaPiabcIcaOiabd6gaUnaaCaaaleqabaGaeGOmaidaaOGaeyOeI0YaaabCaeaacqWGMbGzdaqhaaWcbaGaemyAaKgabaGaeGOmaidaaaqaaiabdMgaPjabg2da9iabicdaWaqaaiabdUgaRbqdcqGHris5aOGaeiykaKcabaGaem4AaSMaemOBa42aaWbaaSqabeaacqaIYaGmaaaaaaaa@481F@

The simplification offered by Cliff [7] is not presented here due to notational differences between his paper and Ferguson's. Both modifications maintain the assumption that items are dichotomous.

Thus specified, *δ *ranges from zero to one. When *δ *= 0.0, the questionnaire has minimal discrimination, and this occurs when all respondents have the same scale score, that is, the questionnaire fails to discriminate any respondent from any other respondent. When *δ *= 1.0, the questionnaire has maximal discrimination since all possible scores occur with the same frequency.

As noted, all current formulae depend on the questionnaire items being dichotomous and coded as 0 or 1. This ensures that all summed scale scores fall within the range 0..*k*, and that the maximum number of different summed scores is *k*+1. Attempts to compute *δ *for polytomous item measures fail because the summed scale scores no longer fall within the range 0..*k*, and the length of the summed scale is no longer fixed at *k*+1. The summed scale range of polytomous item measures will vary according to the *number of response categories *as well as the number of items.

A modified formula to take into account polytomous item measures is presented below. To distinguish the resulting statistic *δ *from the strictly dichotomous form, I suggest appending the subscript *G *(*δ*_*G*_, for Generalised *δ*). Hence when *δ *is cited, it may be assumed that the measure comprises either dichotomous or dichotomised items, and that when *δ*_*G *_is cited, it may be assumed that the measure comprises polytomous items. *δ*_*G *_may be applied to dichotomous scales; the older *δ *may not be applied to polytomous scales.

If we consider a questionnaire scale comprising *k *items with each item having *m *response categories with each item coded 0..*m*-1, the possible range of scores is 0..*k*(*m*-1). For example, a scale comprising 12 items with four responses per item would have a scale range of 0..36, hence:

δG=n2−∑i=0k(m−1)fi2n2−n21+k(m−1)
 MathType@MTEF@5@5@+=feaafiart1ev1aaatCvAUfKttLearuWrP9MDH5MBPbIqV92AaeXatLxBI9gBaebbnrfifHhDYfgasaacH8akY=wiFfYdH8Gipec8Eeeu0xXdbba9frFj0=OqFfea0dXdd9vqai=hGuQ8kuc9pgc9s8qqaq=dirpe0xb9q8qiLsFr0=vr0=vr0dc8meaabaqaciaacaGaaeqabaqabeGadaaakeaaiiGacqWF0oazdaWgaaWcbaGaem4raCeabeaakiabg2da9maalaaabaGaemOBa42aaWbaaSqabeaacqaIYaGmaaGccqGHsisldaaeWbqaaiabdAgaMnaaDaaaleaacqWGPbqAaeaacqaIYaGmaaaabaGaemyAaKMaeyypa0JaeGimaadabaGaem4AaSMaeiikaGIaemyBa0MaeyOeI0IaeGymaeJaeiykaKcaniabggHiLdaakeaacqWGUbGBdaahaaWcbeqaaiabikdaYaaakiabgkHiTmaalaaabaGaemOBa42aaWbaaSqabeaacqaIYaGmaaaakeaacqaIXaqmcqGHRaWkcqWGRbWAcqGGOaakcqWGTbqBcqGHsislcqaIXaqmcqGGPaqkaaaaaaaa@5222@

Where: *n *= sample size

*f *= frequency of score *i*

*k *= number of questionnaire items

*m *= length of scale

Modifying the simplified equation (2):

δG=(1+k(m−1))(n2−∑i=0k(m−1)fi2)n2k(m−1)
 MathType@MTEF@5@5@+=feaafiart1ev1aaatCvAUfKttLearuWrP9MDH5MBPbIqV92AaeXatLxBI9gBaebbnrfifHhDYfgasaacH8akY=wiFfYdH8Gipec8Eeeu0xXdbba9frFj0=OqFfea0dXdd9vqai=hGuQ8kuc9pgc9s8qqaq=dirpe0xb9q8qiLsFr0=vr0=vr0dc8meaabaqaciaacaGaaeqabaqabeGadaaakeaaiiGacqWF0oazdaWgaaWcbaGaem4raCeabeaakiabg2da9maalaaabaGaeiikaGIaeGymaeJaey4kaSIaem4AaSMaeiikaGIaemyBa0MaeyOeI0IaeGymaeJaeiykaKIaeiykaKIaeiikaGIaemOBa42aaWbaaSqabeaacqaIYaGmaaGccqGHsisldaaeWbqaaiabdAgaMnaaDaaaleaacqWGPbqAaeaacqaIYaGmaaGccqGGPaqkaSqaaiabdMgaPjabg2da9iabicdaWaqaaiabdUgaRjabcIcaOiabd2gaTjabgkHiTiabigdaXiabcMcaPaqdcqGHris5aaGcbaGaemOBa42aaWbaaSqabeaacqaIYaGmaaGccqWGRbWAcqGGOaakcqWGTbqBcqGHsislcqaIXaqmcqGGPaqkaaaaaa@5861@

Note that for dichotomous items, *m *= 2 and so *k*(*m*-1) = *k*. Hence for dichotomous items *δ*_*G *_= *δ*.

The modified formula for *δ*_*G *_has been implemented in the statistical software package R as function *delta.g *with 95% confidence limits bootstrapped by resampling with replacement [see Additional file 1]. For those researchers without access to R, a simple spreadsheet is available to compute coefficient *δ*_*G *_from frequency tables. This may be obtained from the author and further implementations are being developed for other platforms.

Having derived the general formula for *δ *it should prove useful to demonstrate the calculation and properties of the coefficient. The 2004 British Household Panel Survey [8] sampled 14761 individuals from the general population (sampling details, protocol and data are available at the survey website [9]). As part of this survey respondents completed the 12-item General Health Questionnaire, a self-report measure of psychiatric morbidity. The data were obtained for an ongoing study of the measurement properties of the GHQ-12 in a general UK sample (usage ID: 21697) and are used here for demonstration purposes only.

The GHQ-12 comprises twelve statements (items) with four responses per item and may be scored dichotomously (0011) or polytomously (0123) [5]. From equation (3), for dichotomous scoring, *k *= 12 and *m *= 2 and for polytomous scoring, *k *= 12 and *m *= 4. There has been much debate over the relative benefits of these and other coding schemes, principally over the establishment of threshold values for clinical severity, but for the purposes of this discussion I will focus on the effect of scoring method on reliability and discrimination. To this end, the reliability of each scoring method was estimated using Cronbach's *α *and the discrimination by *δ*_*G*_.

## Results

**Figure 1:**
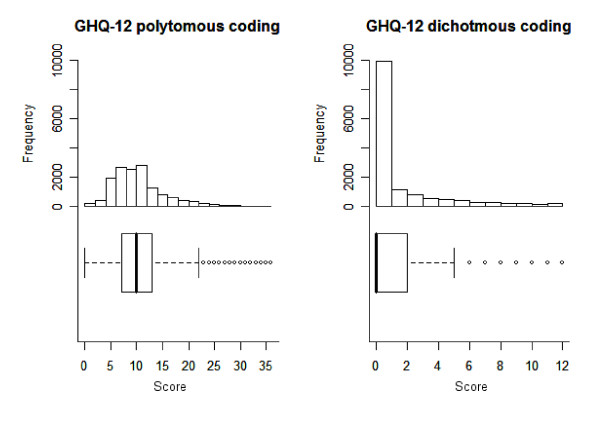
Frequency histograms and boxplots for GHQ-12 dichotomous and polytomous codings (N = 14761).

As can be seen in Figure 1, the distribution of the GHQ-12 score was greatly affected by the scoring method. Polytomous coding produced a slightly skewed distribution but one with clearly defined tails (skew = 1.3, SE = 0.02), with discrimination *δ*_*G *_= 0.96 (actual value: 0.957; bootstrapped 95% CL: 0.956, 0.959) and reliability of *α *= 0.88. Dichotomous coding resulted in a highly-skewed distribution (skew = 1.86, SE = 0.02) with 54.2% of the sample scoring the scale minimum: this lack of discrimination was reflected in the value of *δ*_*G *_= 0.73 (actual value: 0.731; bootstrapped 95% CL: 0.723, 0.739). Reliability was *α *= 0.89. The two scoring methods were highly correlated (*r *= 0.90, p < 0.001).

## Discussion

The results demonstrate the utility of *δ*_*G *_in distinguishing between discriminating and undiscriminating questionnaires. In terms of reliability the two scoring methods were indistinguishable, since Cronbach's alpha was 0.88 for polytomous scoring and 0.89 for dichotomous coding. We would conclude on this basis that the scales were equally reliable. The two methods yielded highly correlated scores (*r *= 0.9): this implies that the coding method did not greatly affect the validity of measurement since the two methods would be likely to correlate equally well with any external criterion.

Consideration of *δ*_*G *_would, however, lead us to a different conclusion, since dichotomous coding produced a scale with a lower index of discrimination (*δ*_*G *_= 0.73) than polytomous coding (*δ*_*G *_= 0.96). Dichotomous coding substantially reduced the ability of the GHQ-12 to distinguish between individuals compared to the four-point coding. Both coding methods resulted in a skewed distribution, but the dichotomous coding resulted in more than half of the sample scoring the same (zero): in effect the questionnaire could not distinguish any difference between these cases. Hence, the discrimination of the questionnaire was compromised, and the degree to which it was compromised was quantified by *δ*_*G*_.

## Conclusion

This paper attempts to reintroduce coefficient *δ *as an index of questionnaire discrimination. The coefficient is non-parametric, making no assumptions of the data, and is conceptually simple, being the ratio of observed discriminations to the maximum possible number of discriminations. The general form *δ*_*G *_is useful for the evaluation and design of the majority of questionnaire measures, that is, those comprising several items with the same number of response categories. It is simple to further modify the formula to take into account scales comprising items with different numbers of responses, such as the SF-36. The statistic may also be used for single-item measures.

It is hoped that researchers will now report and seek to maximise both coefficients of reliability and discrimination when evaluating and designing questionnaire measures. Consideration of the discrimination of a questionnaire should lead to an improvement in the quality of measurement: this should result in greater understanding of the characteristics of different questionnaires in different populations, and also allow questionnaires to be compared and selected on characteristics other than reliability.

The comparative neglect of Ferguson's *δ *and its lack of generality have resulted in an absence of studies to elucidate its sampling distribution and other characteristics, in particular its relationship to validity, reliability and effect size. Further studies of these characteristics will be forthcoming.

## Competing interests

The author(s) declare that they have no competing interests.

## Authors' contributions

MH is the sole author.

## Pre-publication history

The pre-publication history for this paper can be accessed here:



## Supplementary Material

Additional File 1 R code and examples. The file contains R code for calculating coefficient delta and bootstrapped 95% confidence limits. Click here for file
