# Supplementary material for: Questionnaire discrimination: (re)-introducing coefficient δ
Source: BMC Med Res Methodol. 2007 May 18;7:19. doi: 10.1186/1471-2288-7-19 (PMC1884165; doi:10.1186/1471-2288-7-19)
Supplement: Additional File 1 — R code and examples. The file contains R code for calculating coefficient delta and bootstrapped 95% confidence limits. [file 1471-2288-7-19-S1.doc]

R code for delta.g

delta.g<-function(i,k,m)

{

f<-table(i)

nsq<-sum(f)^2

sumfsq<-sum(f^2)

km<-k*(m-1)

d<-(km+1)*(nsq-sumfsq)/(km*nsq)

d

}

Function: computes generalised delta (*δ_G_*)

Usage: delta.g(i,k,m)

Where: i is the variable name

k is the number of items in the scale

m is the number of item responses

Examples:

If the GHQ-12 is scored dichotomously (0011) and summed to variable ghq_d, then k = 12 and m = 2:

> delta.g(ghq_d,12,2)

[1] 0.7313853

If the GHQ-12 is scored polytomously (0123) and summed to variable ghq_l, then k = 12 and m = 4:

> delta.g(ghq_l,12,4)

[1] 0.957903

If p1 a *single item* of the scale ghq_l, then k = 1 and m = 4:

> delta.g(p1,1,4)

[1] 0.5294231

To compute delta-g for ghq_l with item p1 deleted:

> delta.g(ghq_l-p1,11,4)

[1] 0.9567124

R code for bootstrapped 95%CL of delta.g

boot.delta.g<-function(i,k,n) {

boot<-numeric(1000)

for (j in 1:1000) boot[j]<-deltag(sample(i,replace=T),k,n)

d<-delta.g(i,k,n)

bd<-mean(boot)

ucl<-quantile(boot,0.975)

lcl<-quantile(boot,0.025)

a<-structure(c(d,bd,lcl,ucl),.Names=c("Delta","Bootstrapped", "Lower 95%CL","Upper 95%CL"))

a}

Function: Computes delta.g and bootstrapped delta.g with 95%CL.

Bootstrap is 1000 resamplings with replacement.

Bootstrapped delta.g is mean value over 1000 samples.

95% confidence limits are lower and upper 2.5% quantiles.

Usage: boot.delta.g(i,k,m)

Where: i is the variable name

k is the number of items in the scale

m is the number of item responses

Examples:

> boot.delta.g(ghq_d,12,2)

Delta Bootstrapped Lower 95%CL Upper 95%CL

0.7313853 0.7314468 0.7233219 0.7392722

> boot.delta.g(ghq_l,12,4)

Delta Bootstrapped Lower 95%CL Upper 95%CL

0.9579030 0.9578125 0.9567398 0.9588528

> boot.delta.g(p1,1,4)

Delta Bootstrapped Lower 95%CL Upper 95%CL

0.5294231 0.5291585 0.5174977 0.5404438

> boot.delta.g(ghq12l-p1,11,4)

Delta Bootstrapped Lower 95%CL Upper 95%CL

0.9567124 0.9566420 0.9554707 0.9577228
